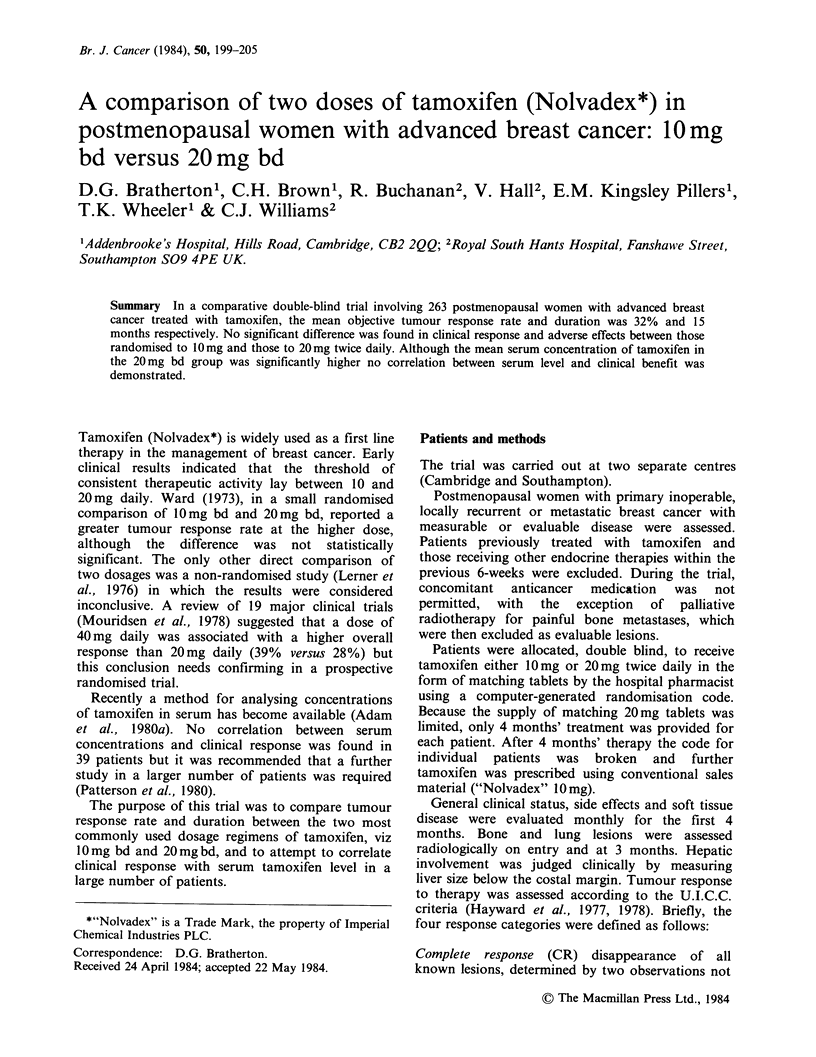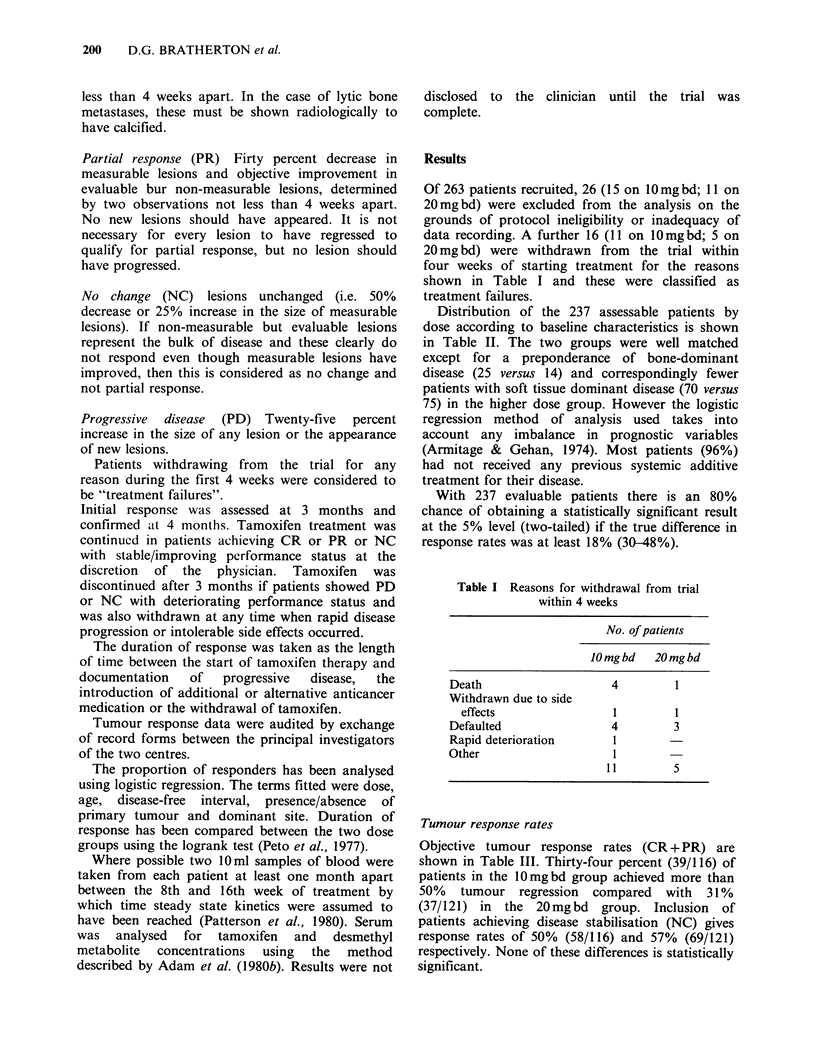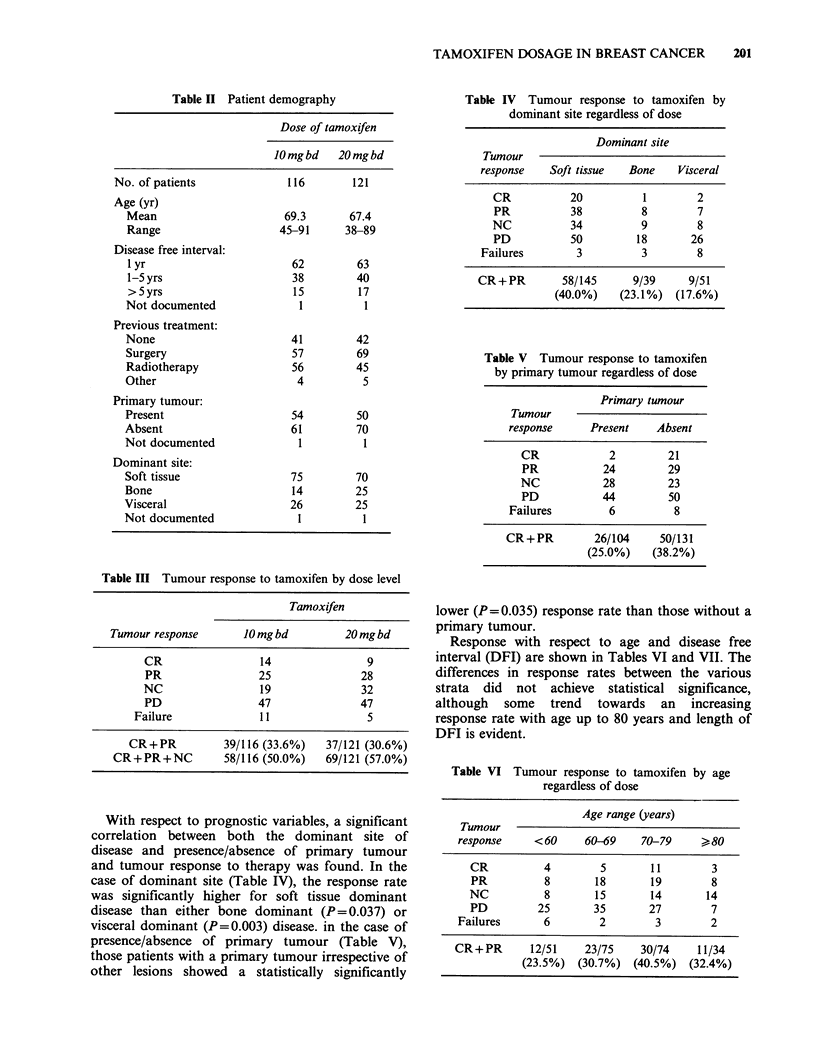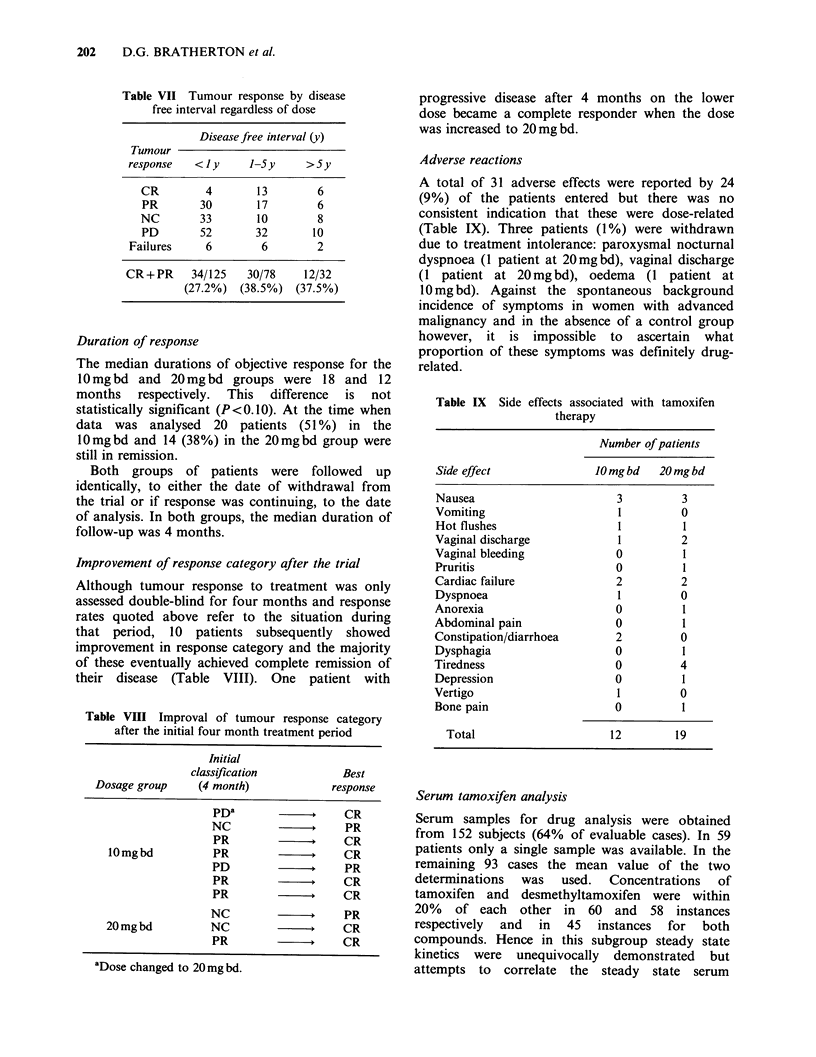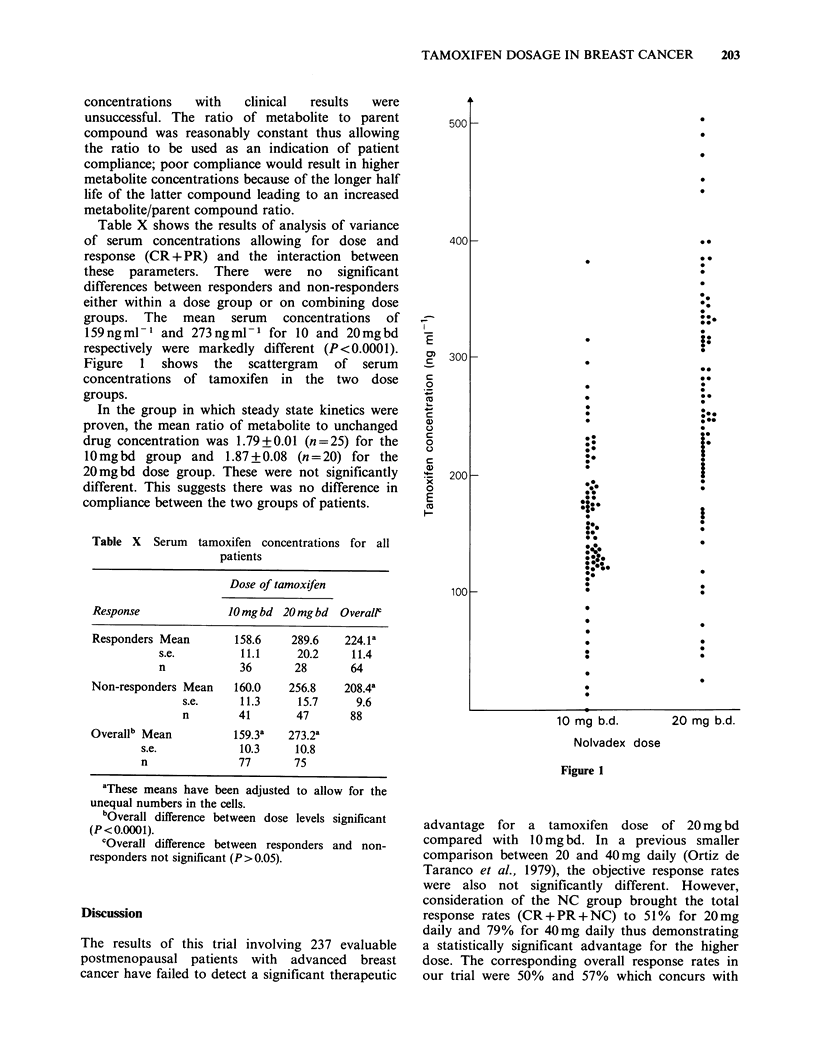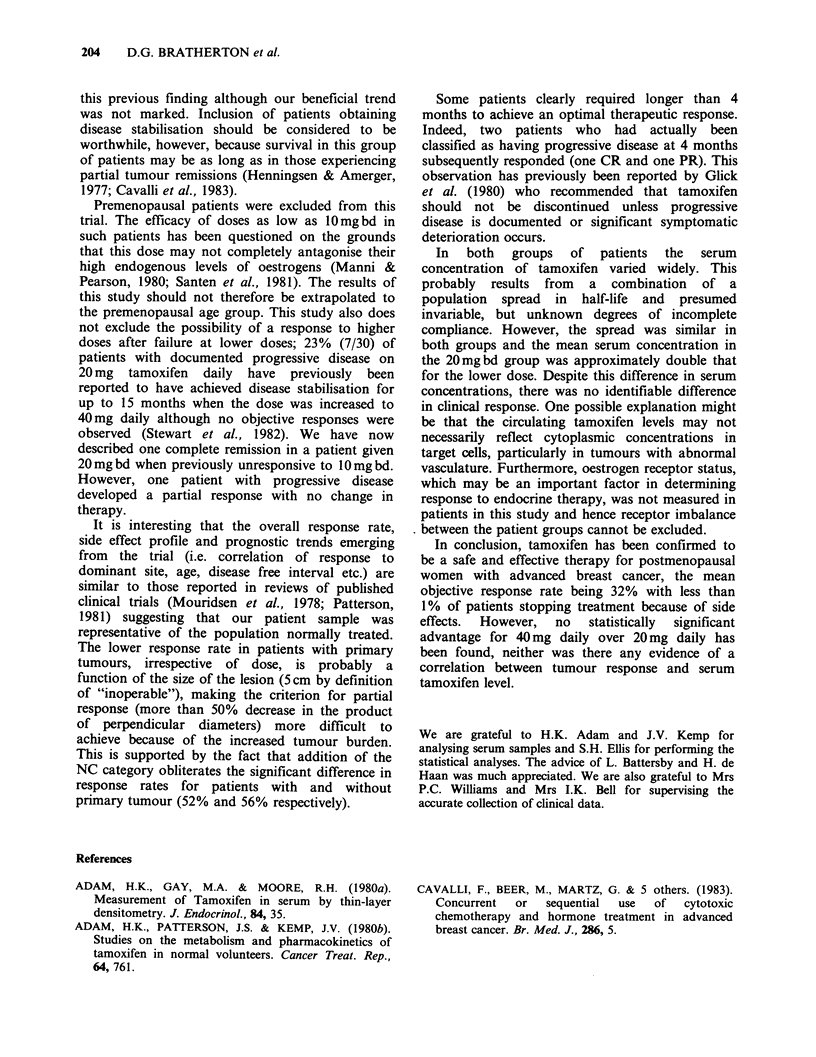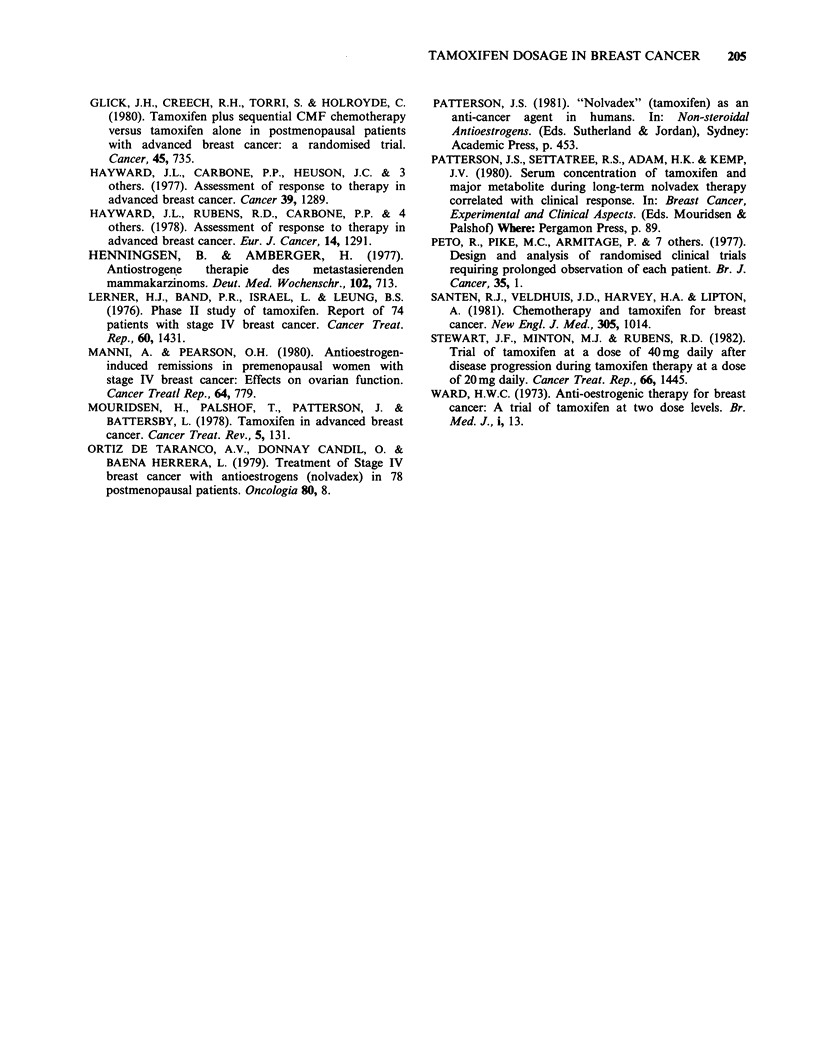# A comparison of two doses of tamoxifen (Nolvadex) in postmenopausal women with advanced breast cancer: 10 mg bd versus 20 mg bd.

**DOI:** 10.1038/bjc.1984.163

**Published:** 1984-08

**Authors:** D. G. Bratherton, C. H. Brown, R. Buchanan, V. Hall, E. M. Kingsley Pillers, T. K. Wheeler, C. J. Williams

## Abstract

In a comparative double-blind trial involving 263 postmenopausal women with advanced breast cancer treated with tamoxifen, the mean objective tumour response rate and duration was 32% and 15 months respectively. No significant difference was found in clinical response and adverse effects between those randomised to 10 mg and those to 20 mg twice daily. Although the mean serum concentration of tamoxifen in the 20 mg bd group was significantly higher no correlation between serum level and clinical benefit was demonstrated.


*Br. J. Cancer* (1984), **50**, 199–205

### A comparison of two doses of tamoxifen (Nolvadex*) in postmenopausal women with advanced breast cancer: 10 mg bd versus 20 mg bd

D.G. Bratherton[1], C.H. Brown[1], R. Buchanan[2], V. Hall[2], E.M. Kingsley Pillers[1], T.K. Wheeler[1] & C.J. Williams[2]

[1]*Addenbrooke's Hospital, Hills Road, Cambridge, CB2 2QQ*; [2]*Royal South Hants Hospital, Fanshawe Street, Southampton SO9 4PE UK.*

**Summary** In a comparative double-blind trial involving 263 postmenopausal women with advanced breast cancer treated with tamoxifen, the mean objective tumour response rate and duration was 32% and 15 months respectively. No significant difference was found in clinical response and adverse effects between those randomised to 10 mg and those to 20 mg twice daily. Although the mean serum concentration of tamoxifen in the 20 mg bd group was significantly higher no correlation between serum level and clinical benefit was demonstrated.

Tamoxifen (Nolvadex*) is widely used as a first line therapy in the management of breast cancer. Early clinical results indicated that the threshold of consistent therapeutic activity lay between 10 and 20 mg daily. Ward (1973), in a small randomised comparison of 10 mg bd and 20 mg bd, reported a greater tumour response rate at the higher dose, although the difference was not statistically significant. The only other direct comparison of two dosages was a non-randomised study (Lerner *et al.*, 1976) in which the results were considered inconclusive. A review of 19 major clinical trials (Mouridsen *et al.*, 1978) suggested that a dose of 40 mg daily was associated with a higher overall response than 20 mg daily (39% *versus* 28%) but this conclusion needs confirming in a prospective randomised trial.

Recently a method for analysing concentrations of tamoxifen in serum has become available (Adam *et al.*, 1980*a*). No correlation between serum concentrations and clinical response was found in 39 patients but it was recommended that a further study in a larger number of patients was required (Patterson *et al.*, 1980).

The purpose of this trial was to compare tumour response rate and duration between the two most commonly used dosage regimens of tamoxifen, viz 10 mg bd and 20 mg bd, and to attempt to correlate clinical response with serum tamoxifen level in a large number of patients.

*"Nolvadex" is a Trade Mark, the property of Imperial Chemical Industries PLC.

Correspondence: D.G. Bratherton.

Received 24 April 1984; accepted 22 May 1984.

### Patients and methods

The trial was carried out at two separate centres (Cambridge and Southampton).

Postmenopausal women with primary inoperable, locally recurrent or metastatic breast cancer with measurable or evaluable disease were assessed. Patients previously treated with tamoxifen and those receiving other endocrine therapies within the previous 6-weeks were excluded. During the trial, concomitant anticancer medication was not permitted, with the exception of palliative radiotherapy for painful bone metastases, which were then excluded as evaluable lesions.

Patients were allocated, double blind, to receive tamoxifen either 10 mg or 20 mg twice daily in the form of matching tablets by the hospital pharmacist using a computer-generated randomisation code. Because the supply of matching 20 mg tablets was limited, only 4 months' treatment was provided for each patient. After 4 months' therapy the code for individual patients was broken and further tamoxifen was prescribed using conventional sales material ("Nolvadex" 10 mg).

General clinical status, side effects and soft tissue disease were evaluated monthly for the first 4 months. Bone and lung lesions were assessed radiologically on entry and at 3 months. Hepatic involvement was judged clinically by measuring liver size below the costal margin. Tumour response to therapy was assessed according to the U.I.C.C. criteria (Hayward *et al.*, 1977, 1978). Briefly, the four response categories were defined as follows:

*Complete response* (CR) disappearance of all known lesions, determined by two observations not

© The Macmillan Press Ltd., 1984

less than 4 weeks apart. In the case of lytic bone metastases, these must be shown radiologically to have calcified.

*Partial response* (PR) Firty percent decrease in measurable lesions and objective improvement in evaluable bur non-measurable lesions, determined by two observations not less than 4 weeks apart. No new lesions should have appeared. It is not necessary for every lesion to have regressed to qualify for partial response, but no lesion should have progressed.

*No change* (NC) lesions unchanged (i.e. 50% decrease or 25% increase in the size of measurable lesions). If non-measurable but evaluable lesions represent the bulk of disease and these clearly do not respond even though measurable lesions have improved, then this is considered as no change and not partial response.

*Progressive disease* (PD) Twenty-five percent increase in the size of any lesion or the appearance of new lesions.

Patients withdrawing from the trial for any reason during the first 4 weeks were considered to be "treatment failures".
Initial response was assessed at 3 months and confirmed at 4 months. Tamoxifen treatment was continued in patients achieving CR or PR or NC with stable/improving performance status at the discretion of the physician. Tamoxifen was discontinued after 3 months if patients showed PD or NC with deteriorating performance status and was also withdrawn at any time when rapid disease progression or intolerable side effects occurred.

The duration of response was taken as the length of time between the start of tamoxifen therapy and documentation of progressive disease, the introduction of additional or alternative anticancer medication or the withdrawal of tamoxifen.

Tumour response data were audited by exchange of record forms between the principal investigators of the two centres.

The proportion of responders has been analysed using logistic regression. The terms fitted were dose, age, disease-free interval, presence/absence of primary tumour and dominant site. Duration of response has been compared between the two dose groups using the logrank test (Peto *et al.*, 1977).

Where possible two 10 ml samples of blood were taken from each patient at least one month apart between the 8th and 16th week of treatment by which time steady state kinetics were assumed to have been reached (Patterson *et al.*, 1980). Serum was analysed for tamoxifen and desmethyl metabolite concentrations using the method described by Adam *et al.* (1980*b*). Results were not disclosed to the clinician until the trial was complete.

### Results

Of 263 patients recruited, 26 (15 on 10 mg bd; 11 on 20 mg bd) were excluded from the analysis on the grounds of protocol ineligibility or inadequacy of data recording. A further 16 (11 on 10 mg bd; 5 on 20 mg bd) were withdrawn from the trial within four weeks of starting treatment for the reasons shown in Table I and these were classified as treatment failures.

Distribution of the 237 assessable patients by dose according to baseline characteristics is shown in Table II. The two groups were well matched except for a preponderance of bone-dominant disease (25 *versus* 14) and correspondingly fewer patients with soft tissue dominant disease (70 *versus* 75) in the higher dose group. However the logistic regression method of analysis used takes into account any imbalance in prognostic variables (Armitage & Gehan, 1974). Most patients (96%) had not received any previous systemic additive treatment for their disease.

With 237 evaluable patients there is an 80% chance of obtaining a statistically significant result at the 5% level (two-tailed) if the true difference in response rates was at least 18% (30–48%).

**Table I** Reasons for withdrawal from trial within 4 weeks

| | *No. of patients* | |
|---|---|---|
| | *10 mg bd* | *20 mg bd* |
| Death | 4 | 1 |
| Withdrawn due to side effects | 1 | 1 |
| Defaulted | 4 | 3 |
| Rapid deterioration | 1 | — |
| Other | 1 | — |
| | 11 | 5 |

### *Tumour response rates*

Objective tumour response rates (CR+PR) are shown in Table III. Thirty-four percent (39/116) of patients in the 10 mg bd group achieved more than 50% tumour regression compared with 31% (37/121) in the 20 mg bd group. Inclusion of patients achieving disease stabilisation (NC) gives response rates of 50% (58/116) and 57% (69/121) respectively. None of these differences is statistically significant.

**Table II** Patient demography

| | Dose of tamoxifen | |
|---|---|---|
| | *10 mg bd* | *20 mg bd* |
| No. of patients | 116 | 121 |
| Age (yr) | | |
| Mean | 69.3 | 67.4 |
| Range | 45–91 | 38–89 |
| Disease free interval: | | |
| 1 yr | 62 | 63 |
| 1–5 yrs | 38 | 40 |
| >5 yrs | 15 | 17 |
| Not documented | 1 | 1 |
| Previous treatment: | | |
| None | 41 | 42 |
| Surgery | 57 | 69 |
| Radiotherapy | 56 | 45 |
| Other | 4 | 5 |
| Primary tumour: | | |
| Present | 54 | 50 |
| Absent | 61 | 70 |
| Not documented | 1 | 1 |
| Dominant site: | | |
| Soft tissue | 75 | 70 |
| Bone | 14 | 25 |
| Visceral | 26 | 25 |
| Not documented | 1 | 1 |

**Table III** Tumour response to tamoxifen by dose level

| | *Tamoxifen* | |
|---|---|---|
| *Tumour response* | *10 mg bd* | *20 mg bd* |
| CR | 14 | 9 |
| PR | 25 | 28 |
| NC | 19 | 32 |
| PD | 47 | 47 |
| Failure | 11 | 5 |
| CR+PR | 39/116 (33.6%) | 37/121 (30.6%) |
| CR+PR+NC | 58/116 (50.0%) | 69/121 (57.0%) |

With respect to prognostic variables, a significant correlation between both the dominant site of disease and presence/absence of primary tumour and tumour response to therapy was found. In the case of dominant site (Table IV), the response rate was significantly higher for soft tissue dominant disease than either bone dominant ($P=0.037$) or visceral dominant ($P=0.003$) disease. in the case of presence/absence of primary tumour (Table V), those patients with a primary tumour irrespective of other lesions showed a statistically significantly lower ($P=0.035$) response rate than those without a primary tumour.

**Table IV** Tumour response to tamoxifen by dominant site regardless of dose

| *Tumour response* | *Dominant site* | | |
|---|---|---|---|
| | *Soft tissue* | *Bone* | *Visceral* |
| CR | 20 | 1 | 2 |
| PR | 38 | 8 | 7 |
| NC | 34 | 9 | 8 |
| PD | 50 | 18 | 26 |
| Failures | 3 | 3 | 8 |
| CR+PR | 58/145 (40.0%) | 9/39 (23.1%) | 9/51 (17.6%) |

**Table V** Tumour response to tamoxifen by primary tumour regardless of dose

| *Tumour response* | *Primary tumour* | |
|---|---|---|
| | *Present* | *Absent* |
| CR | 2 | 21 |
| PR | 24 | 29 |
| NC | 28 | 23 |
| PD | 44 | 50 |
| Failures | 6 | 8 |
| CR+PR | 26/104 (25.0%) | 50/131 (38.2%) |

Response with respect to age and disease free interval (DFI) are shown in Tables VI and VII. The differences in response rates between the various strata did not achieve statistical significance, although some trend towards an increasing response rate with age up to 80 years and length of DFI is evident.

**Table VI** Tumour response to tamoxifen by age regardless of dose

| *Tumour response* | *Age range (years)* | | | |
|---|---|---|---|---|
| | *<60* | *60–69* | *70–79* | *≥80* |
| CR | 4 | 5 | 11 | 3 |
| PR | 8 | 18 | 19 | 8 |
| NC | 8 | 15 | 14 | 14 |
| PD | 25 | 35 | 27 | 7 |
| Failures | 6 | 2 | 3 | 2 |
| CR+PR | 12/51 (23.5%) | 23/75 (30.7%) | 30/74 (40.5%) | 11/34 (32.4%) |

**Table VII** Tumour response by disease free interval regardless of dose

| Tumour response | Disease free interval (y) | | |
|---|---|---|---|
| | <1 y | 1–5 y | >5 y |
| CR | 4 | 13 | 6 |
| PR | 30 | 17 | 6 |
| NC | 33 | 10 | 8 |
| PD | 52 | 32 | 10 |
| Failures | 6 | 6 | 2 |
| CR+PR | 34/125 (27.2%) | 30/78 (38.5%) | 12/32 (37.5%) |

*Duration of response*

The median durations of objective response for the 10 mg bd and 20 mg bd groups were 18 and 12 months respectively. This difference is not statistically significant ($P<0.10$). At the time when data was analysed 20 patients (51%) in the 10 mg bd and 14 (38%) in the 20 mg bd group were still in remission.

Both groups of patients were followed up identically, to either the date of withdrawal from the trial or if response was continuing, to the date of analysis. In both groups, the median duration of follow-up was 4 months.

*Improvement of response category after the trial*

Although tumour response to treatment was only assessed double-blind for four months and response rates quoted above refer to the situation during that period, 10 patients subsequently showed improvement in response category and the majority of these eventually achieved complete remission of their disease (Table VIII). One patient with progressive disease after 4 months on the lower dose became a complete responder when the dose was increased to 20 mg bd.

**Table VIII** Improval of tumour response category after the initial four month treatment period

| Dosage group | Initial classification (4 month) | | Best response |
|---|---|---|---|
| 10 mg bd | PD[a] | ⟶ | CR |
| | NC | ⟶ | PR |
| | PR | ⟶ | CR |
| | PR | ⟶ | CR |
| | PD | ⟶ | PR |
| | PR | ⟶ | CR |
| | PR | ⟶ | CR |
| 20 mg bd | NC | ⟶ | PR |
| | NC | ⟶ | CR |
| | PR | ⟶ | CR |

[a]Dose changed to 20 mg bd.

*Adverse reactions*

A total of 31 adverse effects were reported by 24 (9%) of the patients entered but there was no consistent indication that these were dose-related (Table IX). Three patients (1%) were withdrawn due to treatment intolerance: paroxysmal nocturnal dyspnoea (1 patient at 20 mg bd), vaginal discharge (1 patient at 20 mg bd), oedema (1 patient at 10 mg bd). Against the spontaneous background incidence of symptoms in women with advanced malignancy and in the absence of a control group however, it is impossible to ascertain what proportion of these symptoms was definitely drug-related.

**Table IX** Side effects associated with tamoxifen therapy

| Side effect | Number of patients | |
|---|---|---|
| | 10 mg bd | 20 mg bd |
| Nausea | 3 | 3 |
| Vomiting | 1 | 0 |
| Hot flushes | 1 | 1 |
| Vaginal discharge | 1 | 2 |
| Vaginal bleeding | 0 | 1 |
| Pruritis | 0 | 1 |
| Cardiac failure | 2 | 2 |
| Dyspnoea | 1 | 0 |
| Anorexia | 0 | 1 |
| Abdominal pain | 0 | 1 |
| Constipation/diarrhoea | 2 | 0 |
| Dysphagia | 0 | 1 |
| Tiredness | 0 | 4 |
| Depression | 0 | 1 |
| Vertigo | 1 | 0 |
| Bone pain | 0 | 1 |
| Total | 12 | 19 |

*Serum tamoxifen analysis*

Serum samples for drug analysis were obtained from 152 subjects (64% of evaluable cases). In 59 patients only a single sample was available. In the remaining 93 cases the mean value of the two determinations was used. Concentrations of tamoxifen and desmethyltamoxifen were within 20% of each other in 60 and 58 instances respectively and in 45 instances for both compounds. Hence in this subgroup steady state kinetics were unequivocally demonstrated but attempts to correlate the steady state serum concentrations with clinical results were unsuccessful. The ratio of metabolite to parent compound was reasonably constant thus allowing the ratio to be used as an indication of patient compliance; poor compliance would result in higher metabolite concentrations because of the longer half life of the latter compound leading to an increased metabolite/parent compound ratio.

Table X shows the results of analysis of variance of serum concentrations allowing for dose and response (CR+PR) and the interaction between these parameters. There were no significant differences between responders and non-responders either within a dose group or on combining dose groups. The mean serum concentrations of 159 ng ml$^{-1}$ and 273 ng ml$^{-1}$ for 10 and 20 mg bd respectively were markedly different ($P<0.0001$). Figure 1 shows the scattergram of serum concentrations of tamoxifen in the two dose groups.

In the group in which steady state kinetics were proven, the mean ratio of metabolite to unchanged drug concentration was $1.79 \pm 0.01$ ($n=25$) for the 10 mg bd group and $1.87 \pm 0.08$ ($n=20$) for the 20 mg bd dose group. These were not significantly different. This suggests there was no difference in compliance between the two groups of patients.

**Table X** Serum tamoxifen concentrations for all patients

| *Response* | | *Dose of tamoxifen* 10 mg bd | 20 mg bd | *Overall*[c] |
|---|---|---|---|---|
| Responders | Mean | 158.6 | 289.6 | 224.1[a] |
| | s.e. | 11.1 | 20.2 | 11.4 |
| | n | 36 | 28 | 64 |
| Non-responders | Mean | 160.0 | 256.8 | 208.4[a] |
| | s.e. | 11.3 | 15.7 | 9.6 |
| | n | 41 | 47 | 88 |
| Overall[b] | Mean | 159.3[a] | 273.2[a] | |
| | s.e. | 10.3 | 10.8 | |
| | n | 77 | 75 | |

[a]These means have been adjusted to allow for the unequal numbers in the cells.

[b]Overall difference between dose levels significant ($P<0.0001$).

[c]Overall difference between responders and non-responders not significant ($P>0.05$).

**Figure 1**

### Discussion

The results of this trial involving 237 evaluable postmenopausal patients with advanced breast cancer have failed to detect a significant therapeutic advantage for a tamoxifen dose of 20 mg bd compared with 10 mg bd. In a previous smaller comparison between 20 and 40 mg daily (Ortiz de Taranco *et al.*, 1979), the objective response rates were also not significantly different. However, consideration of the NC group brought the total response rates (CR+PR+NC) to 51% for 20 mg daily and 79% for 40 mg daily thus demonstrating a statistically significant advantage for the higher dose. The corresponding overall response rates in our trial were 50% and 57% which concurs with this previous finding although our beneficial trend was not marked. Inclusion of patients obtaining disease stabilisation should be considered to be worthwhile, however, because survival in this group of patients may be as long as in those experiencing partial tumour remissions (Henningsen & Amerger, 1977; Cavalli *et al.*, 1983).

Premenopausal patients were excluded from this trial. The efficacy of doses as low as 10 mg bd in such patients has been questioned on the grounds that this dose may not completely antagonise their high endogenous levels of oestrogens (Manni & Pearson, 1980; Santen *et al.*, 1981). The results of this study should not therefore be extrapolated to the premenopausal age group. This study also does not exclude the possibility of a response to higher doses after failure at lower doses; 23% (7/30) of patients with documented progressive disease on 20 mg tamoxifen daily have previously been reported to have achieved disease stabilisation for up to 15 months when the dose was increased to 40 mg daily although no objective responses were observed (Stewart *et al.*, 1982). We have now described one complete remission in a patient given 20 mg bd when previously unresponsive to 10 mg bd. However, one patient with progressive disease developed a partial response with no change in therapy.

It is interesting that the overall response rate, side effect profile and prognostic trends emerging from the trial (i.e. correlation of response to dominant site, age, disease free interval etc.) are similar to those reported in reviews of published clinical trials (Mouridsen *et al.*, 1978; Patterson, 1981) suggesting that our patient sample was representative of the population normally treated. The lower response rate in patients with primary tumours, irrespective of dose, is probably a function of the size of the lesion (5 cm by definition of "inoperable"), making the criterion for partial response (more than 50% decrease in the product of perpendicular diameters) more difficult to achieve because of the increased tumour burden. This is supported by the fact that addition of the NC category obliterates the significant difference in response rates for patients with and without primary tumour (52% and 56% respectively).

Some patients clearly required longer than 4 months to achieve an optimal therapeutic response. Indeed, two patients who had actually been classified as having progressive disease at 4 months subsequently responded (one CR and one PR). This observation has previously been reported by Glick *et al.* (1980) who recommended that tamoxifen should not be discontinued unless progressive disease is documented or significant symptomatic deterioration occurs.

In both groups of patients the serum concentration of tamoxifen varied widely. This probably results from a combination of a population spread in half-life and presumed invariable, but unknown degrees of incomplete compliance. However, the spread was similar in both groups and the mean serum concentration in the 20 mg bd group was approximately double that for the lower dose. Despite this difference in serum concentrations, there was no identifiable difference in clinical response. One possible explanation might be that the circulating tamoxifen levels may not necessarily reflect cytoplasmic concentrations in target cells, particularly in tumours with abnormal vasculature. Furthermore, oestrogen receptor status, which may be an important factor in determining response to endocrine therapy, was not measured in patients in this study and hence receptor imbalance between the patient groups cannot be excluded.

In conclusion, tamoxifen has been confirmed to be a safe and effective therapy for postmenopausal women with advanced breast cancer, the mean objective response rate being 32% with less than 1% of patients stopping treatment because of side effects. However, no statistically significant advantage for 40 mg daily over 20 mg daily has been found, neither was there any evidence of a correlation between tumour response and serum tamoxifen level.

We are grateful to H.K. Adam and J.V. Kemp for analysing serum samples and S.H. Ellis for performing the statistical analyses. The advice of L. Battersby and H. de Haan was much appreciated. We are also grateful to Mrs P.C. Williams and Mrs I.K. Bell for supervising the accurate collection of clinical data.

### References

ADAM, H.K., GAY, M.A. & MOORE, R.H. (1980*a*). Measurement of Tamoxifen in serum by thin-layer densitometry. *J. Endocrinol.*, **84**, 35.

ADAM, H.K., PATTERSON, J.S. & KEMP, J.V. (1980*b*). Studies on the metabolism and pharmacokinetics of tamoxifen in normal volunteers. *Cancer Treat. Rep.*, **64**, 761.

CAVALLI, F., BEER, M., MARTZ, G. & 5 others. (1983). Concurrent or sequential use of cytotoxic chemotherapy and hormone treatment in advanced breast cancer. *Br. Med. J.*, **286**, 5.

GLICK, J.H., CREECH, R.H., TORRI, S. & HOLROYDE, C. (1980). Tamoxifen plus sequential CMF chemotherapy versus tamoxifen alone in postmenopausal patients with advanced breast cancer: a randomised trial. *Cancer*, **45**, 735.

HAYWARD, J.L., CARBONE, P.P., HEUSON, J.C. & 3 others. (1977). Assessment of response to therapy in advanced breast cancer. *Cancer* **39**, 1289.

HAYWARD, J.L., RUBENS, R.D., CARBONE, P.P. & 4 others. (1978). Assessment of response to therapy in advanced breast cancer. *Eur. J. Cancer*, **14**, 1291.

HENNINGSEN, B. & AMBERGER, H. (1977). Antiostrogene therapie des metastasierenden mammakarzinoms. *Deut. Med. Wochenschr.*, **102**, 713.

LERNER, H.J., BAND, P.R., ISRAEL, L. & LEUNG, B.S. (1976). Phase II study of tamoxifen. Report of 74 patients with stage IV breast cancer. *Cancer Treat. Rep.*, **60**, 1431.

MANNI, A. & PEARSON, O.H. (1980). Antioestrogen-induced remissions in premenopausal women with stage IV breast cancer: Effects on ovarian function. *Cancer Treatl Rep.*, **64**, 779.

MOURIDSEN, H., PALSHOF, T., PATTERSON, J. & BATTERSBY, L. (1978). Tamoxifen in advanced breast cancer. *Cancer Treat. Rev.*, **5**, 131.

ORTIZ DE TARANCO, A.V., DONNAY CANDIL, O. & BAENA HERRERA, L. (1979). Treatment of Stage IV breast cancer with antioestrogens (nolvadex) in 78 postmenopausal patients. *Oncologia* **80**, 8.

PATTERSON, J.S. (1981). "Nolvadex" (tamoxifen) as an anti-cancer agent in humans. In: *Non-steroidal Antioestrogens*. (Eds. Sutherland & Jordan), Sydney: Academic Press, p. 453.

PATTERSON, J.S., SETTATREE, R.S., ADAM, H.K. & KEMP, J.V. (1980). Serum concentration of tamoxifen and major metabolite during long-term nolvadex therapy correlated with clinical response. In: *Breast Cancer, Experimental and Clinical Aspects*. (Eds. Mouridsen & Palshof) **Where:** Pergamon Press, p. 89.

PETO, R., PIKE, M.C., ARMITAGE, P. & 7 others. (1977). Design and analysis of randomised clinical trials requiring prolonged observation of each patient. *Br. J. Cancer*, **35**, 1.

SANTEN, R.J., VELDHUIS, J.D., HARVEY, H.A. & LIPTON, A. (1981). Chemotherapy and tamoxifen for breast cancer. *New Engl. J. Med.*, **305**, 1014.

STEWART, J.F., MINTON, M.J. & RUBENS, R.D. (1982). Trial of tamoxifen at a dose of 40 mg daily after disease progression during tamoxifen therapy at a dose of 20 mg daily. *Cancer Treat. Rep.*, **66**, 1445.

WARD, H.W.C. (1973). Anti-oestrogenic therapy for breast cancer: A trial of tamoxifen at two dose levels. *Br. Med. J.*, **i**, 13.